# Metastases of OSCC Based on Oral Lichen Ruber Planus

**DOI:** 10.3390/cancers15164092

**Published:** 2023-08-14

**Authors:** Katharina Theresa Obermeier, Sabina Noreen Wuersching, Paris Liokatis, Wenko Smolka, Philipp Poxleitner, Christin Kleye, Michael Ehrenfeld, Maximilian Kollmuss, Sven Otto

**Affiliations:** 1 Department of Oral and Maxillofacial Surgery and Facial Plastic Surgery, University Hospital, Ludwig Maximilian University Munich, 80337 Munich, Germany; paris.liokatis@med.uni-muenchen.de (P.L.); wenko.smolka@med.uni-muenchen.de (W.S.); philipp.poxleitner@med.uni-muenchen.de (P.P.);; 2 Department of Conservative Dentistry and Periodontology, University Hospital, Ludwig Maximilian University Munich, Goethestrasse 70, 80336 Munich, Germany; sabina.wuersching@med.uni-muenchen.de (S.N.W.);

**Keywords:** oral lichen ruber, OSCC, neck dissection, lymph nodal spreading, second metachronous tumor, metastases

## Abstract

**Simple Summary:**

Oral lichen ruber planus is a poorly understood chronic inflammatory disease of the oral mucosa and can cause malignant transformation into oral squamous cell carcinoma. This study is to our knowledge the first study examining the behavior of lymph nodal spreading in OSCC based on OLP and compares it to OSCC without OLP. Mostly, OSCC with OLP are highly differentiated tumors and come with a lower risk factor for lymph nodal spreading. Secondar metachronous tumor appears more often in patients suffering from OSCC based on OLP than in the comparison group. This study aims to evaluate the oncological characteristics of OSCC with OLP and compare them to a group with OSCC without OLP.

**Abstract:**

Oral lichen ruber planus (OLP) is a poorly understood chronically inflammatory disease of the oral mucosa. Malignant transformation into oral squamous cell carcinoma (OSCC) is reported in between 1–2% of cases in the literature. After malignant transformation, surgical treatment—meaning tumor resection combined with neck dissection—is recommended. The recommended extent of treatment is controversial in the literature because this kind of OSCC is often a highly differentiated tumor with a lower risk for lymph nodal spreading. This study aims to overview 103 patients treated in our department due to OLP. The primary outcome parameter was the development of metastases in OLP patients compared to a group of OSCC patients without OLP and the comparison of survival in between both groups. Statistical analysis showed a significantly lower risk for patients with OSCC and with OLP for lymph nodal spreading (*p* = 0.013). Patients with OSCC and without OLP had a 4.76-higher risk for lymph nodal spreading. On the other hand, second metachronous tumor occurred more often in patients with OSCC and OLP. Overall, OSCC based on OLP occurs more often in female patients, is more highly differentiated and comes with a lower risk for metastases but has a higher risk for second metachronous tumors. Therefore, special attention should be paid to patients with OSCC based on OLP when planning adjuvant therapy and clinical follow-up. The indication for postoperative radiation should be made cautiously in this case, and clinical controls should be performed more closely due to the risk of recurrent disease or tumors at different locations.

## 1. Introduction

Oral lichen ruber planus (OLP) of the mucosa is a chronic inflammatory disease of the mucosal membrane of the oral cavity [1]. Reticular forms with white keratotic striae are the typical clinical appearance of OLP [2,3]. Global prevalence amounts to around 1.01% with the highest prevalence reported in Europe (about 1.43%) and the lowest in India (0.49%) [4]. OLP is a T-cell-mediated chronic inflammatory disease of the oral mucosa. The etiology of OLP is only partly understood; risk factors such as nicotine abuse and genetic transformation are discussed [5,6]. Histologically, it is described as marked immune cell infiltration [7]. Due to the risk of malignant transformation in oral squamous cell carcinoma (OSCC), patients with OLP must be monitored regularly. Malignant transformation is reported to be between 1% to 2% (0–12.5%) [8]. A recent meta-analysis reported that 1.1% of OLP lesions progress into OSCC, with a higher incidence in smokers, alcohol users and those infected with the hepatitis C virus [9]. Several studies identified OLP of the tongue with a high risk for malignant transformation [10,11]. After transforming into OSCC, tumor resection combined with neck dissection is the recommended treatment. The recommended extent of treatment is controversial in the literature [10] because this kind of OSCC is often a highly differentiated tumor with a lower risk for lymph nodal spreading. However, data on OSCC originating from OLP with long-time follow-up are scarce in the literature. This study aims to overview 103 patients treated at the Department of Oral and Maxillofacial Surgery Munich due to OLP. A particular focus lies on patients with malignant transformation into OSCC. In particular, data and characteristics of OSCC were examined taking into account staging, lymph nodal involvement and survival.

## 2. Materials and Methods

This study was approved by the institutional ethics committee of the Ludwig Maximilian University of Munich, Germany (Munich, Germany, ref. number: 22-0218).

**Figure 1:**
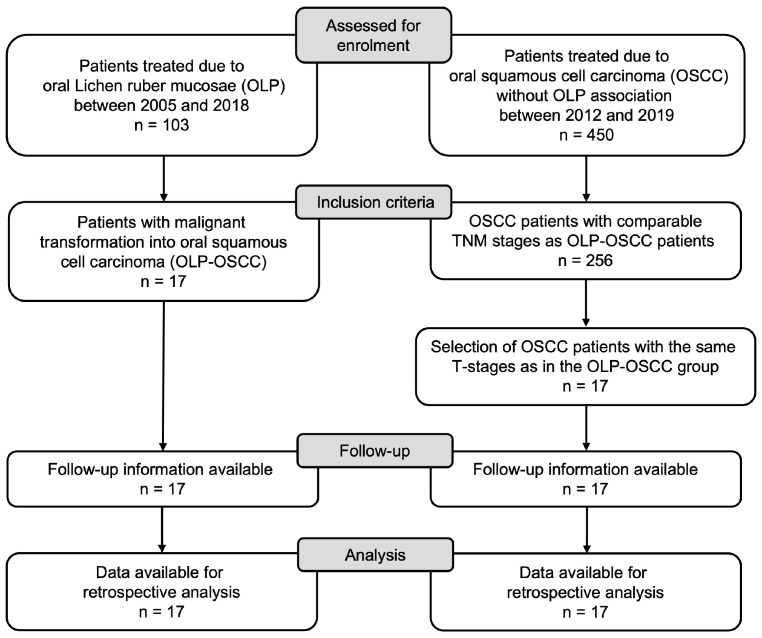
Flow chart.

The present retrospective case-control study includes 103 patients treated due to oral lichen ruber mucosae between 2005–2022 in the department of oral and maxillofacial surgery of LMU Munich. A comparable control group of patients with OSCC without OLP was generated. The comparison group was created from a tumor database of 450 patients. Patients with the same tumor TNM stage were selected. The current AJCC/UICC TNM staging (8th edition, 2017) of OSCC was used for all cases. After selection, 256 patients with comparable tumor staging remained. The flow chart in Figure 1 shows the selection of patients. Seventeen (17) comparable patients with OSCC not based on OLP were randomly selected for the control group. TNM classification, tumor diameter and depth of invasion were considered in order to avoid selection bias when choosing comparable patients. 

### 2.1. Outcome Measures

The primary outcome parameter was the development of metastases in OLP patients compared to a group of OSCC patients without OLP and the comparison of survival between both groups. The secondary outcome parameters were grading, local recurrence and secondary tumor defined as a tumor in a different location.

### 2.2. Exclusion Criteria

Resection status other than R0, meaning complete resection with histologically confirmed safety margin >5 mm, lack of neck dissection and previous history of malignancy or irradiation in the head and neck.

### 2.3. Statistical Analysis

Statistical analysis was conducted using SPSS^®^ 24 version 4.0 (SPSS Inc., Chicago, IL, USA). The Shapiro–Wilk test was used for determining the distribution pattern of the data, and they were found to be not normally distributed. Demographics were reported in a descriptive style. All patients were divided into two groups:

Group A: Patients with OSCC with underlying histopathologically proven OLP
Group B: Patients with OSCC without OLP in the background
Group C: Control group of 255 patients with OSCC without OLP

For calculating the difference between both groups and the frequency of metastases, we used a Chi-square test. Survival analysis was performed by using Kaplan-Meier Analysis, and the Log-Rank-Test was used for comparison of both groups. This was performed in Python 3.8.0 using the packages numpy, lifelines and matplotlib.

Statistical significance was defined as *p* < 0.05. 

## 3. Results

Oncological characteristics and demographics for groups A and B are shown in Table 1. All patients underwent at least one computed tomography scan (CT) for tumor staging and were treated surgically with curative intention according to the recommendation of an interdisciplinary tumor board. The mean follow-up time amounted to 80.52 ± 43.98 months. 

### 3.1. Results of Group A

Of the 103 included patients, 80 were female (77.7%) and 23 (23.3%) were male. The mean age of the patients at first diagnosis was 61.55 ± 11.31 years (range 42–85). Overall malignant transformation was found in 17 patients (16.05%). The average time between the first diagnosis and malignant transformation was 3.1 ± 3.78 (range 6 months 132) years. The mean age at first diagnosis of OSCC amounted to 66.6 ± 11.93 years. Fifteen patients (88.2%) with malignant transformation were female, and two were male (17.5%). In four patients, suspicious lymph nodes were noticed in the preoperative staging examinations.

Overall, lymph nodal spreading was found in 3 patients (17.6%); all three received adjuvant radiation. 

The average tumor diameter amounted to 2.24 ± 1.37 cm. The average depth of invasion was 0.78 ± 0.65 cm. In 9 patients (52.9%), OSCC was graded as well-differentiated, in 7 patients (41.1%) as moderately differentiated and in only 1 patient (5.9%) as poorly differentiated. Bone infiltration was found in 2 cases; one of those patients received adjuvant radiotherapy. In two cases bone infiltration was found in moderately differentiated tumors and in one case in a well-differentiated tumor. 

Two patients (23.5%) suffered from local recurrence. One patient suffered three times from local recurrence, with the first recurrence appearing each year postoperatively. The second patient suffered twice from recurrence, after 12 months and 48 months, respectively. In three patients from group A, a second synchronous tumor in the area of the lichen manifestation was found at primary diagnosis. Five patients suffered from a second tumor with manifestation at a different site in the oral cavity during follow-up at 6, 12, 14, 16 and 21 months, respectively. Second primary tumor occurred on the tongue in three patients, on the alveolar ridge in one case and on the planum buccale in one patient.

During follow-up, one patient developed lymph nodal recurrence three years after primary diagnosis; in this case, a neck dissection was performed.

**Figure 2:**
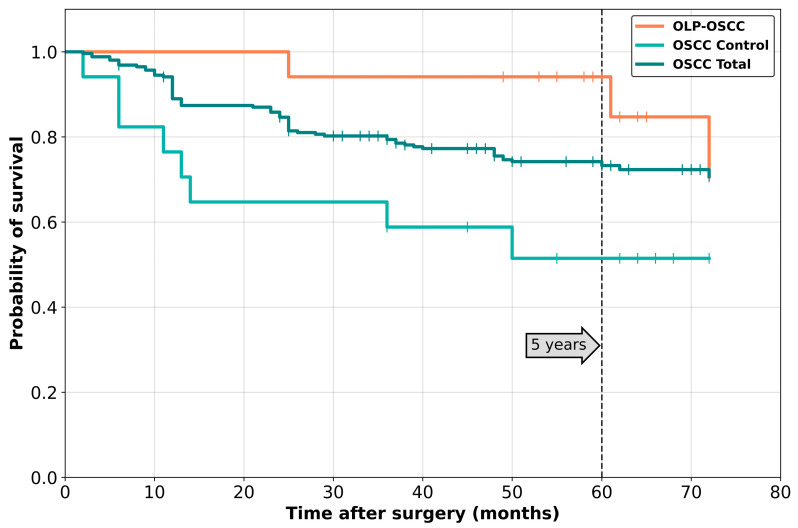
Survival curves of all three groups.

Four patients (23.5%) died after an average time of 75.5 months due to tumor-related disease. The survival curve is shown in Figure 2. 

### 3.2. Results of Group B

Overall, 17 patients suffering from OSCC without OLP were included. Table 1 gives an overview of tumor staging, grading, tumor diameter and depth of invasion.

Average age at first diagnosis of OSCC amounted to 65.1 ± 10.96 years. Nine patients (52.9%) with OSCC were female, and eight were male (47.1%). In six patients, lymph nodes presented suspiciously in the staging CT scan. 

Overall, lymph nodal spreading was found in 10 patients (58.9%). The average tumor diameter amounted to 2.25 ± 1.25 cm. The average depth of invasion amounted to 0.61 ± 0.91 cm. Perineural invasion was not found in any case. Bone infiltration was found in 3 cases. Only in 4 patients (23.5%) was the tumor graded as well-differentiated—which is lower than in group A—in 8 patients (47%) as moderately differentiated and in 5 patients (29.4%) as G3, meaning poorly differentiated. 

One patient suffered from local recurrence. The mean follow-up time was 40.2 ± 25.87 months. Eight patients (47%) died after a mean time of 17.5 months. Two patients died due to cardiovascular complications, and six patients due to tumor disease progression. In group B, no second metachronous tumor was observed.

One patient developed a lymph nodal recurrence that was found one year after primary surgical treatment. 

### 3.3. Results of Group C

Overall survival of group C compared to group A and B is shown in Figure 2.

### 3.4. Results of Statistical Analysis

The Chi-square test showed a significant difference between both groups concerning lymph nodal spreading (*p* = 0.013), meaning the risk for nodal involvement was significantly lower in patients with OSCC with OLP. 

**Figure 3:**
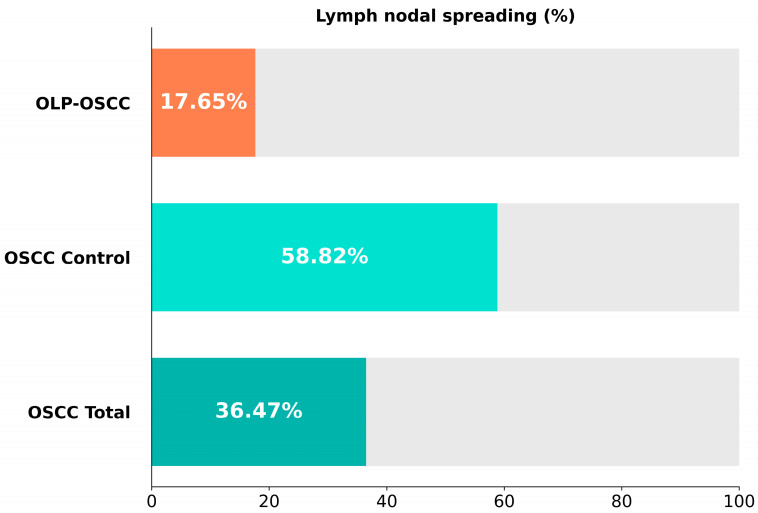
Overview of lymph nodal spreading.

The odds ratio for developing lymph nodal spreading was 4.46 for group B. Due to the low number of patients, there was no statistical difference concerning local recurrence between the two groups. Figure 3 shows the distribution of lymph nodal spreading in group A, B and C. 

Survival analysis showed a statistical significance between both groups (*p* = 0.025). Figure 3 shows the survival curves of both groups. 

## 4. Discussion

OLP is a chronic inflammatory disease of the oral mucosa which can cause symptoms and is challenging in clinical management [12]. Malignant transformation into OSCC is undoubtedly the most severe complication of OLP [13]. Malignant transformation of OLP into OSCC appears in 0–12.5% of patients [1,8]. In our study, malignant transformation was found in 16.05% of patients and is higher than previously reported. This discrepancy may be due to fact that patients who are referred to a highly specialized center for oral oncology typically present more severe forms of the disease. Moreover, the follow-up time in our study was relatively high (84 months) compared to other studies: in the study by Gonzalez-Moles et al. 2012, the follow-up was 12 months and in the one by Zotti et al. 2021, 31.62 months [14,15]. The average time between the first diagnosis of OLP and malignant transformation amounted to 37 months, similar to other studies [16,17]. In our study, 88.2% of the patients suffering from OSCC after OLP were female as OLP predominantly occurs in women [18]. In contrast, the control group had a balanced ratio between females and males (52.9%:41.1%). 

G3 grading (poorly differentiated) was only found in one patient in the OLP group but in 5 cases (29.4%) in group B. A total of 52.9% of OLP-OSCC tumors were classified as well-differentiated and 41.1% as moderately differentiated, whereas in the OSCC group without OLP, only 23.5% were classified as well-differentiated and 47.1% as moderately differentiated. OSCC based on OLP seems to be more highly differentiated in terms of tumor grading. Due to the small population in our study, it remains a topic of discussion as to whether a definitive statement can be made about the distribution of grading for our patient groups where it has been shown that there is a difference in grading. To verify this, however, a larger patient population would have to be considered. Bombeccari et al. 2011 observed similar results in their study: 75% were well-differentiated, 25% were moderately differentiated and none were poorly differentiated [10]. Poor differentiation comes with a higher risk for lymph nodal spreading, which can also explain the high number of metastases. Well-differentiation occurred more often in OSCC based on OLP with a ratio of 9:4 compared to Group B. Poor differentiation OSCC occurred more often in OSCC without OLP (5:1). 

Mignoga et al. 2002 reported an alarming tendency to develop metachronous tumors in patients with OLP-OSCC [19,20]. We report similar findings. In our cohort, three patients (17.6%) developed a second primary OSCC in a different location in the oral cavity during follow-up. Interestingly, there was no topographic relationship between second primary tumors and primary localizations. None of the patients in the OSCC-without-OLP group suffered from a second primary tumor. Therefore, it has to be discussed whether these patients have to receive close diagnostic procedures such as panendoscopy to rule out secondary tumors. On the other hand, there are anesthesiological complications as well as surgical complications, such as esophageal perforation complications, bleeding and dental fractures and, critically, a delay in the start of tumor therapy due to a preceding panendoscopy.

Local recurrence was found more frequently in the OLP-OSCC group. Four patients (23.5%) suffered from local recurrence, one of them three times. In the OSCC group without OLP, only one patient suffered from local recurrence. Margins were clear after each tumor resection in all of these patients with a safety margin of >5 mm. Bombeccari et al. 2011 report local recurrence rates of up to 37.5% in patients with OLP and malignant transformation [10]. The high local recurrence rates for OLP-OSCC may be explained by the chronic inflammatory nature of the OLP, which can facilitate tumor growth in further sites by causing DNA damage in other tissue regions affected by OLP. As a location for OLP, the tongue is known to have a higher risk for malignant transformation [21]. We report similar findings in our patients: 52.9% of our patients with malignant transformation suffered from OLP of the tongue. 

One aim of this study was to examine the frequency of lymph nodal spreading in patients suffering from OSCC after the malignant transformation of OLP. Only 17.6% of all patients suffered from metastases, while lymph nodal spreading was found in 58.9% of the control group. This number exceeds the 30% reported in the literature [22]. This was probably because the tumor stages in the group without OLP should be comparable to those with lichen, and many patients had an advanced tumor stage. Chi-square testing showed statistical significance between the OLP-OSCC group and the comparison group in terms of lymph nodal spreading (*p* = 0.013). The odds ratio for developing metastases in OSCC patients without OLP was 4.76 times higher than in patients suffering from OSCC due to malignant transformation of OLP. Another study reports that none of the patients with malignant transformation of OLP suffered from metastases [10]. 

In contrast, Mignoga et al. 2001 reported patients with OLP and malignant transformation within a population of 4 patients [19]. All of them developed lymph nodal metastases. The authors suggest a more radical treatment for patients suffering from malignant transformation of OLP. Overall survival was significantly higher in group A. Lymph node metastasis is one of the most important prognostic factors for tumor patients and drops survival rates by 50% [23]. In group B, 58.9% of all patients suffered from metastases, which is quite high compared to the literature. In Figure 2, lymph nodal spreading of all three groups is shown. Metastases in the control group (group C) were found in 36.47% of cases, which is similar to the literature. The number of patients with metastases in group B is probably higher in patients matched to patients from group A by tumor stage, size and thickness. Nevertheless, compared to group A, the amount of lymph nodal spreading in group C is higher. The same is shown in Figure 3, where the overall survival of patients from group C is also higher than that of group B. Here, too, the patients were not matched according to tumor size.

Although the results of our study are consistent with other studies, several limitations of our study should be considered. In terms of risk factors, several factors, such as information about alcohol and nicotine consumption, were not evaluated in this study. The present study represents only one geographical region (Southern Germany), and only Caucasian patients were included. However, we included 17 patients with primary diagnosis of OLP and subsequent malignant transformation in the follow-up. Based on the results of this study, it is worth considering whether the treatment of patients with OSCC based on lichen ruber should receive a different type of staging. Due to the increased incidence of local recurrence and especially second tumors in this case, it is worth considering whether these patients should undergo closer clinical follow-ups and, additionally, receive regular panendoscopy during staging. Early detection of a second tumor or recurrence can play an important role in survival as well as in the patient’s quality of life [23]. The risk for cervical lymph node metastases seems lower in this patient group; also, OSCC based on OLP are more highly differentiated tumors; therefore, adjuvant therapy should be discussed in an interdisciplinary setting. In contrast, the risk of metastases is significantly higher for patients in the group with OSCC without OLP. Adjuvant therapy in radiochemotherapy should be considered here, as should regular sonography of the cervical lymph nodes during follow up.

## 5. Conclusions

This study shows that OSCC based on OLP occurs more frequently in women and has a higher risk for second metachronous tumors and lower risk for lymph node metastases. The risk for developing lymph nodal spreading in OSCC with underlying OLP was statistically significantly lower (*p* = 0.013): patients with OSCC without OLP had a 4.76-higher risk for lymph nodal metastases. Also, in this study it was shown that OSCC based on OLP are more high differentiated tumors. Based on the results of this study, it is important to subject patients with OSCC based on OLP to closer clinical follow-ups. Radiotherapy to reduce the risk of lymph node metastases should be discussed in those patients and should be considered interdisciplinary.

## Figures and Tables

**Table 1:** Overview of demographic and oncological data for groups A and B.

	Group: A OSCC with OLP	Group B: OSCC without OLP
Median age (years)	66.6	65.1
Gender		
Male	2 (17.5%)	9 (52.9%)
Female	15 (88.2%)	8 (47.1%)
Tumor Localization		
Mouth floor	0	2
Tongue	9	6
Planum bu.	3	3
Maxilla	1	1
Lip	1	0
Alveolar	3	5
T stadium		
T1	8	8
T2	5	5
T3	1	1
T4a	3	3
N stadium		
cN0	6	0
pN0	8	7
N1	2	3
N2a	0	4
N2b	1	1
Grading		
G1	9	4
G2	7	8
G3	1	5
Neck dissection	11	17
One-sided	5	9
Both-sided	6	8
Local recurrence	2	1
Second tumor	5	0

## Data Availability

The data presented in this study are available in this article.
